# A Novelty Procedure to Identify Critical Causes of Materials Incompatibility

**DOI:** 10.3390/ma16103884

**Published:** 2023-05-22

**Authors:** Dominika Siwiec, Jacek Pacana, Andrzej Pacana

**Affiliations:** Faculty of Mechanical Engineering and Aeronautics, Rzeszow University of Technology, al. Powstancow Warszawy 12, 35-959 Rzeszow, Poland; pacanaj@prz.edu.pl (J.P.); app@prz.edu.pl (A.P.)

**Keywords:** quality, ACO, ant colony optimization, non-destructive testing, incompatibility of material, non-conformities, impact on environment, decision support, industrial products, production engineering, mechanical engineering

## Abstract

Ensuring the expected quality of materials is still a challenge, mainly in order to precisely plan improvement actions that allow for stabilization of the production process. Therefore, the purpose of this research was to develop a novel procedure to identify critical causes of material incompatibility—the causes that have the largest negative impact on material deterioration, and the natural environment. The main originality of this procedure is developing a way to coherent analyse the mutual influence of the many causes of incompatibility of any material, after which the critical causes are identified and a ranking of improvement actions to eliminate these causes is created. A novelty is also developed in the algorithm supporting this procedure, which can be realized in three different ways to solve this problem, i.e.; by considering the impact of material incompatibility on: (i) the deterioration of the material quality; (ii) the deterioration of the natural environment; and (iii) simultaneously the deterioration of the quality of the material and the natural environment. The effectiveness of this procedure was confirmed after tests on 410 alloy, from which a mechanical seal was made. However, this procedure can be useful for any material or industrial product.

## 1. Introduction

Improving the quality of materials and industrial products remains a challenge. It is still difficult to ensure the repeatability of the results under industrial conditions, mainly as part of special material processes [1,2]. It refers to the processing and production of products compatible with quality [3,4], in order for special processes to be realised based on the validated algorithm or method [5,6]. The companies are taking different actions in this area [7]. One of the basic actions is the investigation of the quality of materials and processes according to the qualitative analysis method for materials, products, or production processes [8,9]. Additionally, the stabilisation of the quality of materials and products is supported by qualitative tests, which should be performed according to the continuous improvement of the cycle [6,7,10,11]. It refers to continuously identifying all defects and then taking adequate improvement actions (i.e., eliminating or reducing these defects in the future) [8,12,13,14]. In this area, especially popular in industry practise is, e.g., destructive testing (DT) [15] or non-destructive testing (NDT) [16]. However, the results of these controls still do not show what the main problem is with defects occurring in materials or products [17]. Therefore, it is necessary to conduct further analyses in this regard to adequately undertake the improvement actions mentioned above [18,19], including those that stabilise the quality of materials and industrial products [20,21,22]. Therefore, the objective of the investigation was to develop a procedure to identify critical causes of material incompatibility.

Analysis of the literature on the subject showed that a lot of analysis was carried out using non-destructive or destructive tests, e.g., [15,16,23,24]. After these controls, many conduct analyses using quality management methods and tools. For example, the authors of articles [25,26,27] used the Ishikawa diagram to analyse the potential causes of materials and industrial products. The Ishikawa diagram is called a fishbone diagram (or chevron diagram). As part of the aforementioned research, its use supported the process of identifying defects in materials or products for further analysis. Other popular tools are the Pareto-Lorenz analysis, which the authors of the study used, for example [20,21,22,28,29,30]. Pareto analysis was used to identify the most frequently repeated incompatibilities between materials or industrial products. This is carried out according to the Pareto principle, according to which 20% of all non-conformities generate 80% of the remaining ones. It has been noticed that the Ishikawa diagram is usually combined with the aforementioned Pareto analysis, as, for example, in studies [29,31,32,33]. This combination allows us to identify all incompatibility causes and then select the most important according to the number of times they occur. In turn, the authors of the article [34,35,36] developed methods for analysing the incompatibility of materials or industrial products, in which selected quality management instruments were combined with selected multi-criteria decision methods (MDCM). For example, in article [34], it was the combined brainstorming method (BM), the Ishikawa diagram with the 5M rule, and the DEMATEL method. This method made it possible to analyse the causes of potential non-compliance with the product in order to determine the most important causes affecting its quality. Another example is article [35], in which, for example, the SMART(-ER) method, brainstorming (BM), Ishikawa diagram, Likert scale validation technique, arithmetic average, and Grey Relational Analysis (GRA) are combined [37]. In this approach, the aim was to determine a sequence of actions adequate to the importance of the reasons for the non-conformity of the material or industrial product.

Based on a review of the literature on the subject, it was shown that research was carried out on the identification and elimination of the causes of non-compliance of materials or industrial products. To date, analyses to identify the causes of the main non-conformities have often involved the use of traditional quality management techniques [29,31,32,33], which in some cases were supported by selected decision support techniques [26,34]. Furthermore, previous research was found to focus mainly on qualitative aspects (improvement of the quality of materials and industrial products), but aspects of impact on the natural environment were omitted [30,31,32,38,39,40].

Therefore, the main conclusions regarding the simultaneously identified research gaps concerned a small number of studies that would present methodologies supporting a systematic and consistent qualitative and environmental analysis of non-compliance of materials or products, i.e., the lack of such studies that would ensure the creation of rankings of the causes of non-compliance of materials or industrial products, taking into account their mutual impact on the deterioration of the quality of the material or product as well as their mutual negative impact on the environment. In addition, it was concluded that no methodology supporting the identification of the main causes of non-compliance of materials or processes has been presented so far, so the methodology should be based on analyses of the degree of mutual influence of the causes of potential non-compliance of materials or industrial products in three areas, i.e.,

(i) only for the mutual influence of potential causes on the occurrence of non-conformities (deterioration of the quality of materials or industrial products);
(ii) only for mutual influences of potential causes determining the impact of these causes on the natural environment (deterioration of the natural environment);
(iii) for the simultaneous impact of potential causes on the occurrence of nonconformities and on the environment.

In addition, previous analyses of the incompatibility of materials or industrial products were largely subjective and uncertain [41,42]. This is due to the use of mainly qualitative quality management methods. Additionally, the problem analysis process (i.e., from identifying non-compliances to defining improvement actions) was often laborious and not supported by computer-operated algorithms.

Hence, the aim of the research was to develop a procedure to identify critical causes of material incompatibility. As part of the research, the following hypothesis was adopted:

**Hypothesis** **1.** *Pro-quality improvement of materials and industrial products can be made on the basis of rankings of reasons for non-compliance of these materials and products, where these rankings will be created after a coherent and sequential analysis of the mutual degree of the impact of these causes on: (a) the occurrence of non-conformities (deterioration of the quality of materials and products); (b) and/or the natural environment (negative environmental impact of the causes of potential incompatibility of materials and products)*.

The originality of the developed procedure to identify critical causes of material incompatibility lies mainly in:

- the ability to precisely and effectively analyse the causes of non-compliance identified in any materials and industrial products, where these analyses will be carried out depending on the needs:
  - in order to identify the causes of the main non-conformities causing a significant deterioration of the quality of these materials or industrial products;
  - identify the main causes of non-compliance with materials or industrial products causing a significant deterioration of the natural environment;
  - in order to identify the main causes that will be responsible for both the deterioration of the quality of materials or industrial products and the deterioration of the natural environment;
- determining in a precise and dynamic way (effectively, supported by computer software algorithms) a queue of improvement actions, which will be undertaken only for the most important causes of non-compliance of materials and industrial products.

The procedure test was carried out for the porosity cluster, which was identified by non-destructive testing (fluorescence method) in one of the Polish enterprises. This discrepancy was identified on a new-generation 410 alloy mechanical seal.

## 2. Procedure to Identify Critical Causes of Materials Incompatibility

### 2.1. Concept of Procedure

The concept of the procedure was based on a sequential and coherent process to improve the quality of materials and industrial products, mainly as part of stabilising the quality of new or frequently modified products. The idea of developing the procedure resulted from the need to support decision-making processes, which in general relate to the following: they boil down to solving the following problem. It is assumed that any quality control (e.g., NDT or DT) [15] identifies a non-conformity in a material or an industrial product (e.g., not stabilised in quality).

Incompatibilities (e.g., cracks, notches, or general geometric discontinuities) have a significant impact on material quality. They can also contribute to the formation and deepening of other defects. Depending on the type of inconsistency and the degree of material degradation, they affect, among others [43,44,45]:

- deterioration of mechanical properties;
- change of electrical properties;
- poor state of optical properties;
- deterioration of thermal properties;
- change of magnetic properties;
- affect the arising and/or changing damage;
- performance of the material or product, including its lifetime;
- safety of structural elements;
- electronic band structures of 2D materials.

In turn, the occurrence of an identified non-conformity is usually caused by a large number of probable (potential) causes, the importance (significance) of which is unknown due to the occurrence of the non-conformity. In addition, the potential causes of non-compliance may affect both the deterioration of the quality of the material or industrial product and may have a negative impact on the natural environment.

Therefore, it is problematic to precisely (possibly objective), dynamically (not very time-consuming), and methodically consistently determine which of the potential causes are the most important (main) both due to the deterioration of the quality of the material or industrial product and due to the negative impact on the natural environment. Therefore, the research concept involved the development of a procedure to identify critical causes of material incompatibility. The idea of the procedure concerns taking such improvement actions that will simultaneously improve the quality of materials and industrial products, e.g., as part of stabilising their production process, and will help reduce the negative impact of these causes of non-compliance on the natural environment.

The main benefits of the proposed procedure over conventional proven methods such as Ishikawa’s 6M (Man, Machine, Material, Method, Measurement, and Mother Nature), or Why-Why analysis, are such as:

- increase in the precision and accuracy of decision-making on the causes of the main non-compliances;
- the ability to compare each cause in detail against all others, which increases objectivity;
- determination and analysis of the environmental impact not only in the area of thematic causes related to the impact on the natural environment but also environmental impacts resulting from all identified potential causes belonging to different thematic groups (e.g., 5M);
- validating the causes of potential non-compliance regarding quality (impact on quality) and at the same time regarding the impact on the environment;
- the ability to search for the most beneficial improvement activities according to a coherent and uniform procedure, which is additionally supported by an optimization algorithm.

Other benefits of the procedure are presented in the rest of the article.

### 2.2. Assumptions of Procedure

The procedure was assumed to identify critical causes of material incompatibility. These assumptions were developed on the basis of a review of the literature on the subject and previous research.

- Material and industrial products, including non-conformities identified therein, are unlimited [46];
- The number of causes of potential non-compliance is unlimited [47].
- Potential causes of non-compliance with materials or industrial products are those that are likely to cause non-compliance, but the degree of their impact is usually unknown or clearly defined [13,48,49].
- The main causes of non-compliance of materials or industrial products are those that have the greatest impact on the occurrence of non-compliance (and, in the proposed approach, also on the natural environment) [7,14,50].
- The expert (entity) decides what type of analysis he will conduct, i.e., only for qualitative aspects, only for environmental aspects, or for both quality and environmental aspects [9,51,52].
- the expert (entity) determines the relationship between the significance of mutual influences of potential causes on the occurrence of non-compliance (deterioration of the quality of materials or industrial products) and the importance of mutual influences of these potential causes on the deterioration of the natural environment [53].

According to the assumptions adopted, it is possible to use this procedure to determine the sequence of improvement actions according to specific main reasons in terms of quality and environment.

### 2.3. Characteristics of Procedure

**Figure 1:**
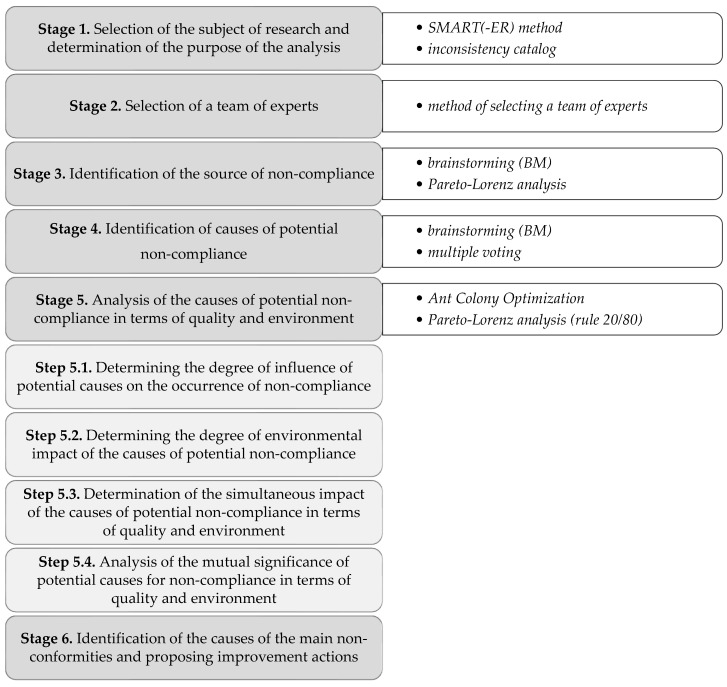
Algorithm of procedure to identify critical causes of material incompatibility.

The procedure was developed in six main stages. To support the process of implementing the stages of the procedure, including making it more dynamic (supported by computer algorithms), selected techniques have been implemented in it. These were mainly quality management tools and machine learning tools, i.e., the SMART(-ER) method [54], brainstorming (BM) [55], method of selecting a team of experts [33,56], Pareto-Lorenz rule (20/80) [30], and Ant Colony Optimization (ACO) [57,58] with MATLAB software. The choice of individual techniques was justified at each stage of the procedure, the algorithm of which is presented in Figure 1.

The description of the procedure is presented in the next part of the article, where the course of action in accordance with the five main stages of the procedure is detailed.

*Step* *1.* *Selection of the subject of research and determination of the purpose of the analysis*

As part of the research, the subject of the analysis should be selected. The choice is made by the entity using the proposed procedure (an expert, a decision-maker, an owner, general director, or a production person). The subject of the analysis should be the product on which the main non-conformity is identified, i.e., the one that most often occurs and generates the greatest losses (waste). It is possible to select such a discrepancy based on a catalogue of discrepancies. This directory is kept relatively often in the company. In the case of a large number of inconsistencies, it is useful to use the Pareto-Lorenz analysis, as presented in the literature, e.g., [28,30]. Subsequently, the purpose of the analysis is defined for the selected research subject. The goal is determined by the subject (an expert). It is possible to use the SMART(-ER) method for this, as presented in the article [54]. In the proposed approach, the goal should be to determine the causes of potential non-compliances, then analyse them sequentially and consistently in order to identify the root causes. On their basis, it is possible to determine the correct (numerically confirmed) direction of improvement for the selected subject of research. Depending on the needs, the purpose may include the type (name, type) of the subject of testing, the non-compliance identified on it, or the number of such non-conformities in a period of time, e.g., on an annual basis. This information is often recorded in the company’s product catalogue.

*Step* *2.* *Selection of a team of experts*

The implementation of the proposed procedure is largely based on the knowledge and experience of experts, i.e., people who have knowledge and competence in the analysed area of research. Hence, the proposed procedure assumes the selection of a team of experts who will be responsible for carrying out the process of improving the quality of materials and industrial products. Following the authors of studies [33,56], to select a team of experts, one should follow the method of selecting a team of experts.

Initially, the entity using the method (an expert) selects n arbitrary experts, mainly paying attention to their knowledge and experience (competence to analyse the selected research problem). It is assumed that each n-selected expert indicates the same number of experts in their group (z = n). Then, the minimum number of experts is estimated according to Equation (1) [33].

(1) $$N>\frac{n\times z\times \left(n-1\right)}{\left(n\times z-\sum_{i=1}^{n}\mu \left(i\right)\right)+1}$$

where: N—required number of experts; z—competent experts selected by n selected experts; $\mu \left(i\right)$—number of non-recurring experts selected by the i-th expert from n groups of experts.

If each expert indicates the same number of experts, it is possible to determine the required number of experts according to Equation (2) [56]:

(2) $$N=\frac{n^{2}\left(n-1\right)}{\left(n^{2}-\sum_{i=1}^{n}\mu \left(i\right)\right)+1}$$

where: N—required number of experts; $\mu \left(i\right)$—number of non-recurring experts selected by the i-th expert from n groups of experts.

Then, the competency coefficient of these experts should be calculated using Equation (3) [33,56]:

(3) $$\begin{array}{ccc} K_{k}=\frac{k_{z}+k_{a}}{2} & where & k_{z}\;and\;k_{a}\epsilon \langle 0;1\rangle \end{array}$$

where: K_k_—expert competence factor; k_z_—coefficient of the degree of knowledge of the problem by the expert; k_a_—argument factor.

The coefficient of degree of knowledge of the problem by the expert (k_z_) and the coefficient of argumentation (k_a_) are determined by the experts themselves. Self-assessment is performed on the basis of the ratings presented in Table 1.

Each expert assesses his knowledge of the problem on a scale of 0 to 10. Subsequently, the points determined by the expert are multiplied by the value of 0.1, creating a value for the coefficient ($k_{z}$) as presented in Formula (4). Later, the argument coefficient is calculated ($k_{a}$), which takes into account the structure of arguments for the award of ratings by individual experts. The higher the $k_{a}$ index, the greater the expert’s practical experience is obtained than the theoretical experience, e.g., $k_{a}$ = 1, $k_{a}$ = 0.75, $k_{a}$ = 0.5—respectively: high—medium—low, the degree of influence of all sources of argument on the opinion of the expert. This is represented by Formula (4). The threshold value of the competence coefficient ($K_{k}$) is equal to 0.6 [56]. Therefore, experts for whom the coefficient $K_{k}$ was less than 0.6, as in Equation (4) cannot be included in the team of experts [33]:

(4) $$\left.\begin{array}{c} \begin{array}{ccc} k_{z}=p\times 0.1 & where & k_{z}\epsilon \langle 0;1\rangle \end{array}\\ \begin{array}{ccc} k_{a}=a_{1}+a_{2}+a_{3} & where & k_{a}\epsilon \langle 0;1\rangle \end{array}\\ T=N-\left(N\epsilon K_{k}<0.6\right) \end{array}\right\}$$

where: p—rating awarded by it-h expert; a—argumentation rating, N—required number of experts.

The obtained number T is the number of experts competent to analyse the verified problem. A leader can be appointed from among the team of experts who will be responsible for coordinating the team’s work. It should be someone who will increase the probability of achieving the research goal.

*Step* *3.* *Identification of the source of non-compliance*

Determining the source of non-compliance consists of determining the place where the non-conformity arose. For this purpose, brainstorming (BM) can be carried out by a team of experts, as presented in the study [43,55]. If a large number of probable places of non-compliance are identified, only the most probable ones should be selected, using, for example, the Pareto-Lorenz analysis [59].

*Step* *4.* *Identification of causes of potential non-compliance*

Initially, all potential causes of nonconformities should be identified, i.e., those that probably contributed to the occurrence of nonconformities. This means answering the question “What happened that the non-compliance occurred?”. These reasons are determined by a team of experts in brainstorming (BM). To this end, a team of experts identifies as many causes as possible. Each should be written down in a visible place, e.g., a board. This process takes a maximum of 30 min. Then, from all identified causes, unrealistic causes should be removed, i.e., those that practically have no impact on the occurrence of non-compliance [55]. In contrast, for all practical reasons, an aggregate list is created. Based on the list of these potential causes, the next stage of the procedure is implemented.

*Step* *5.* *Analysis of the causes of potential non-compliance in terms of quality and environment*

It was assumed that the proposed procedure, depending on the needs, could be applied in three ways, i.e.,

(i) Only for the degree of impact of potential causes, determining the impact of these causes on the occurrence of nonconformities;
(ii) only for the degree of impact of potential causes, determining the impact of these causes on the natural environment;
(iii) for the simultaneous impact of potential causes on the occurrence of nonconformities and on the environment.

If, for the entity using the proposed procedure, only the quality of the product or material is important, then the procedure should be carried out only for step 5.1. If only the environmental impact is significant, then only step 5.2 must be followed. However, if both the quality of the product or material and its impact on the environment are important, then the entire procedure from step 5.1 to step 5.4 should be followed.

In addition, depending on the preferences of the entity (expert) using the proposed procedure, it is possible to control the ratio of the impact of the causes of nonconformity on the quality of the product to the impact of these causes on the natural environment. This is done by validating the grades awarded in the appropriate weight ratio. The selection of the importance of the quality-environment relationship depends on the needs of the expert analysis, as presented in the further stages of the procedure.

Step 5.1. Determining the degree of influence of potential causes on the occurrence of non-compliance

Determining the degree of impact of potential causes on the occurrence of nonconformities results from the fact that potential causes can be subjectively determined to have a greater or lesser impact on the occurrence of nonconformities. A proper determination of this impact will ensure a consistent and more precise assessment of these causes in order to correctly select the root causes (those having the greatest impact on the occurrence of the non-conformity). Following the authors of the papers, it was assumed that this impact is determined by dividing the number of points among all potential causes from the list (no more than 50 points for a given cause). The higher the number of points, the lesser the cause of the discrepancy. The mutual importance of potential causes for the occurrence of non-conformities is determined on the basis of their degree of mutual influence. This is implemented in a decision matrix for pairwise comparisons. The rows and columns of the matrix are the individual potential causes of non-compliance and the corresponding values of the degree of impact of these causes. The pairwise comparison of the degree of influence of the causes of non-conformity is to calculate the difference between the mutual influences of these causes, as presented in Equation (5) [46,60]:

(5) $$a_{ij}^{k}=\left|b_{i}^{k}-b_{j}^{k}\right|$$

where: b—the value of the degree of impact of the cause of non-conformity k, determining its degree of impact on the occurrence of non-conformity or on the natural environment; k—the cause of non-conformity of the product; i, j—1, 2, 3…, n.

Depending on the preferences of the subject (expert), using the proposed procedure, it is possible to control the ratio of the impact of the causes of non-conformity on the quality of the product to the impact of these causes on the natural environment. If quality is as important as environmental impact (1:1), then the values assigned so far remain unchanged and the modelling should continue. Otherwise, you can multiply the assigned quality values by their assumed importance. For example, if quality is 80% important in relation to quality, then the values from the qualitative matrix should be multiplied by 0.8 [28]. The importance of the quality-environment relationship depends on the needs of the expert’s analysis.

Next, the data from the matrix should be prepared for further analysis using ACO [57]. This consists of modifying the matrix so that its first row and column contain values for the reasons that have the greatest cumulative mutual influence on the occurrence of inconsistencies. Therefore, the cumulative mutual influence of the relationship between the causes of potential non-conformities should then be determined. For this purpose, all values of $a_{ij}^{k}$ should be summed up separately for each cause and recorded in a separate column as “total impact of mutual relations of potential causes on the occurrence of non-conformities”. Then, determine the maximum value from these total values. The potential cause with this value has the greatest total mutual influence on the occurrence of discrepancies; therefore, the values from the matrix for this cause should be replaced with the values from the first row and first column of the matrix. Then it is possible to start the correct ACO analysis.

Step 5.2. Determining the degree of environmental impact of the causes of potential non-compliance

Then, it was assumed to determine the degree of negative impact of potential causes on the natural environment. This is due to the need to take action towards sustainable development of production, i.e., so as to effectively take into account environmental, economic, and social aspects. In this case, it is primarily the determination of environmental aspects, i.e., the relationship between the occurrence of the causes of non-compliance and the negative impact of their occurrence on the environment. In this way, it is possible to carry out further analysis in terms of quality and environment, i.e., at the same time paying attention to the criteria that deteriorate the quality of the product and the criteria that have a negative impact on the natural environment. As in the previous step, the environmental impact of potential causes is assessed by a team of experts. For this purpose, experts distribute no more than 50 points between individual potential causes in the list [61]. The higher the number of points, the less impact the cause has on the environment. The mutual importance of potential causes for the occurrence of non-conformities is determined on the basis of their degree of mutual influence. As before, this is done in a decision matrix for pairwise comparisons. The rows and columns of the matrix are the individual potential causes of non-compliance and the corresponding values of the degree of impact of these causes. The pairwise comparison of the degree of influence of the causes of non-conformity is to calculate the difference between the mutual influences of these causes, as in Formula (5).

Depending on the preferences of the subject (expert), using the proposed procedure, it is possible to control the ratio of the impact of the causes of non-conformity on the quality of the product to the impact of these causes on the natural environment. This is done in terms similar to those in step 5.1.

Then, as in the previous step, the data from the matrix should be prepared for further analysis using ACO [57]. This consists of modifying the matrix so that its first row and column contain values for the cause having the greatest total mutual impact on the natural environment. Therefore, the cumulative mutual influence of the relationship between the causes of potential non-compliances on the natural environment should then be determined. For this purpose, all values of $a_{ij}^{k}$ should be summed up separately for each cause and recorded in a separate column as “total impact of mutual relations of potential causes on the natural environment”. Then, determine the maximum value from these total values. A potential cause with this value has the greatest total mutual environmental impact, so replace the matrix values for this cause with the values from the first row and the first column of the matrix. Then it is possible to start the correct ACO analysis.

Step 5.3. Determination of the simultaneous impact of the causes of potential non-compliance in terms of quality and environment

In order to carry out a comprehensive analysis of the qualitative and environmental impact of the causes of potential non-compliances, it is necessary to combine the matrix developed in step 5.1 with the matrix developed in step 5.2. To do this, create a new third decision matrix. This matrix is to contain the sum of the values from the matrix containing the values of the degree of mutual influence of given causes on the occurrence of non-compliance with the values of the matrix containing the degree of mutual influence of these causes on the natural environment. Then, the data from the matrix should be prepared for further analysis using ACO.

As indicated in the previous steps, all the values of $a_{ij}^{k}$ should be summed up separately for each cause and written in a separate column as “the total impact of the mutual relations of potential causes in terms of quality and environment. Then, determine the maximum value from these total values. A potential cause with this value has the greatest cumulative mutual impact on the occurrence of nonconformity and on the environment. Therefore, if necessary, the values from the matrix should be replaced for this reason with the values from the first row and first column of the matrix. Then it is possible to start the correct ACO analysis.

Step 5.4. Analysis of the mutual significance of potential causes for non-compliance in terms of quality and environment

According to the proposed procedure, it is possible to analyse the degree of impact of the causes of potential nonconformities regarding quality (impact on non-conformities) and environment (impact on the natural environment). This is due to the difficulty in precisely determining the significance of potential causes in qualitative and environmental terms, which is usually caused by the following:

- a large number of potential causes;
- a different ranking of potential causes in the case of the impact of these causes on the occurrence of non-conformities;
- different ranking of potential causes in the case of the impact of these causes on the natural environment.

ACO, the values from the developed decision matrices should be analysed. According to the adopted research methodology, an ACO analysis should be carried out based on a decision matrix developed for:

(i) the degree of impact of potential causes, determining the impact of these causes on the occurrence of non-conformities;
(ii) the degree of impact of potential causes, determining the impact of these causes on the natural environment;
(iii) simultaneous impact of potential causes on the occurrence of non-conformities (in terms of quality) and on the natural environment.

The first ACO analysis for case (i) should be performed on the basis of data from the impact matrix of the causes of potential nonconformities (step 5.1). The second ACO analysis for case (ii) should be performed on the basis of data from the environmental impact matrix of the causes of potential nonconformities (step 5.2). The third ACO analysis for case (iii) should be performed on the basis of data from a matrix containing the values of the simultaneous impact of the causes of potential non-conformities on the quality of the material or product and on the environment (step 5.3).

Due to the need to define the distances between the significance (importance) of potential causes in qualitative and environmental terms, this task was considered a traveling salesman problem, i.e., an optimization task consisting of determining the minimum (shortest) path to solve the problem. In this case, it is the determination of the queue (ranking) of potential causes, both in terms of their impact on the occurrence of non-conformities and their impact on the natural environment, and then combining the obtained results into a quality-environmental ranking (queue). A dynamic way of analysing the causes of non-compliance is possible according to ant colony optimisation (ACO).

The choice of ACO in this procedure resulted from its benefits, i.e., [62,63,64,65]:

- versatility, i.e., the possibility of using similar types of analyses (similar types of problems, in this case different material inconsistencies);
- robustness, i.e., the ability to apply small changes to similar optimization problems, e.g., searching for causes of non-compliance for the same type of defects;
- efficiency even for small data sets (compared to, e.g., PSO and GA);
- the possibility of combining ACO with other techniques within one procedure methodology;
- effectiveness in analyzing complex problems (problems affected by various factors);
- the ability to save individual analyses;
- quick indication of the most optimal solution, e.g., comparing it to PSO;
- efficient in solving discrete problems;
- relatively uncomplicated compared to other optimisation algorithms.

In ant colony optimisation (ACO), the principle is as follows: each of the ants leaves a pheromone in the correct pheromone variables that correspond to the edges of the path they travelled. These edges are more attractive to other ants. Later, the ants die [66]. In this algorithm, the pairing of pheromones alternates with the activity of the ants. Therefore, the amount of pheromone $\tau_{ij}\left(t\right)$ left on a given edge $\left(i,j\right)$ represents the attractiveness of the path from point i to point j along this edge. Pheromone information changes along with the experience gained while solving the problem [57].

However, the amount of pheromone is proportional to the quality of the path they have marked out, i.e., the shorter the path, the more pheromone. Therefore, it is possible for the ants to move in the right direction. Additionally, pheromone evaporation avoids situations where all ants follow the same path. It is assumed that the ants remember k places visited, but also places to go. The ant determines the graph of the state space and therefore finds the most advantageous route to travel [58,67]. Ants follow the designated path according to the decision matrix $A_{i}={\left[a_{j}^{i}\left(t\right)\right]}_{N_{i}}$, which is created according to the node i created from the local values of the pheromone trail and the local heuristic values, as shown by Equation (6) [57,58,68]:

(6) $$a_{ij}\left(t\right)=\frac{\tau_{ij}\left(t\right){\left[\eta_{ij}\right]}^{\beta }}{\sum_{l\in N_{i}}\tau_{ij}\left(t\right){\left[\eta_{ij}\right]}^{\beta }}\forall_{j}\in N_{i},$$

where: $\tau_{ij}\left(t\right)$—amount of pheromone at the edge$\left(i,j\right)$ in moment t, ηij=1dij—heuristic shift value from point i to point j, $N_{i}$—the set of nearby (adjacent) points to point i, β—coefficient determining the degree of influence of heuristic values.

The probability with which an ant k decides to move from point i to point $j\in N_{i}^{k}$ as part of building a road in the t-th iteration of the algorithm is given by Equation (7) [66,68]:

(7) $$p_{ij}^{k}\left(t\right)=\frac{a_{ij}\left(t\right)}{\sum_{l\in N_{i}^{k}}a_{ij}\left(t\right)},$$

where: $N_{i}^{k}\subseteq N_{i}$—set of nodes near the node i and not visited by the ant k (where the memory of the ant $k-M^{k}$ allows you to select nodes in $N_{i}^{k}$ from the set $N_{i}$).

After the ants have travelled all the paths, it is possible to evaporate the pheromone that is on all the edges. Afterwards, each ant k leaves the pheromone $∆\tau_{ij}^{k}\left(t\right)$ on the edges it has visited (8) [66,67,68]:

(8) $$∆\tau_{ij}^{k}\left(t\right)=\left\{\begin{array}{cc} \frac{q_{ij}}{L^{k}\left(t\right)} & \begin{array}{cc} when & \left(i,j\right)\in T^{k}\left(t\right) \end{array}\\ 0 & \begin{array}{cc} when & \left(i,j\right)\notin T^{k}\left(t\right) \end{array} \end{array}\right.,$$

where: $T^{k}\left(t\right)$—the path found by the ant k in iteration t, $L^{k}\left(t\right)$—length of the found road, q—dose factor of the pheromone left by the ant.

Therefore, it can be concluded that the value of $∆\tau_{ij}^{k}\left(t\right)$ depends on the length of the path travelled by the ant, i.e., the shorter the length of the path, the greater the amount of pheromone.

The process of evaporation and pheromone leaving by ants is carried out for all edges, as shown in Equation (9) [66,69]:

(9) $$\begin{array}{cc} \tau_{ij}\left(t\right)\leftarrow \gamma \tau_{ij}\left(t\right)+∆\tau_{ij}\left(t\right) & \begin{array}{cc} where & ∆\tau_{ij}\left(t\right)=\sum_{k=1}^{m}∆\tau_{ij}^{k}\left(t\right) \end{array} \end{array},$$

where: m—number of ants in each iteration, $\gamma \in \left(\left.0,1\right]\right.$.

In order to dynamically analyse the causes of inconsistencies, it is possible to implement the data in MATLAB software, as presented by the author of the study [60,68]. Then the calculation process is effective and less complicated.

The result of this analysis is a ranking (queue) of non-compliance causes from the most significant to the least significant, where this significance concerns the impact on the occurrence of non-compliance and then the impact on the natural environment. This classification is used in the next stage of the procedure.

*Step* *6.* *Identification of the causes of the main non-conformities and proposing improvement actions*

The purpose of this stage is to determine the causes of the main non-compliances, i.e., those that should be eliminated in the first place in order to significantly: (i) improve the quality of the product; (ii) reduce the negative impact on the natural environment; (iii) simultaneously improve the quality of the product and reduce the negative impact on the natural environment. This is due to the need for research, the methodology of which is determined by the expert (entity) at the fifth stage of the procedure. Depending on the adopted procedure, at this stage, it is possible to analyse the results:

(i) a ranking of potential causes, specifying only the impact of these causes on the occurrence of non-conformities;
(ii) a ranking of potential causes, specifying only the environmental impact of those causes;
(iii) a ranking of potential causes, specifying the simultaneous impact of potential causes on the occurrence of nonconformities and on the environment.

With g a given ranking of the reasons for non-compliance with a material or product, it is possible to determine the causes of the main non-conformities. This involves the use of the Pareto-Lorenz analysis with the 20/80 rule, as presented in articles, e.g., [30]. In the proposed approach, 20% of the top causes in the ranking are root causes. For them, improvement actions should be taken in the first place. Then it is possible to significantly improve the quality of the product and reduce its negative impact on the natural environment. The final decision on possible improvement actions belongs to the entity (expert, broker) and may depend on the production and financial capabilities, or the possibility of correlating various modifications of the criteria states with each other.

## 3. Results

*Step* *1.* *Selection of the subject of research and determination of the purpose of the analysis*

**Figure 2:**
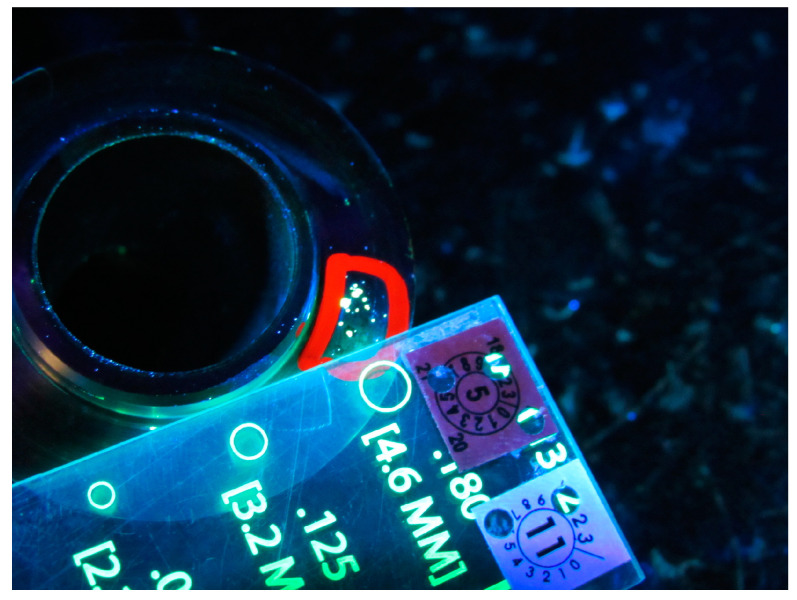
Example of a porosity cluster on a mechanical seal.

The tests were carried out for the popular and commonly identified incompatibility of the material, which is porosity (the occurrence of numerous small gas bubbles located in a group with a random geometric distribution). The subject of research, in which this discrepancy was identified relatively often, was a new-generation mechanical seal made of alloy 410. A mechanical seal is a cast, welded product that allows the fluid to be blocked, inter alia, by a pump or mixer. In this product, the rotating shaft passes through a stationary housing. Consequently, the contact surface between the rotating part and the stationary part is sealed. One of these parts is fixed, but also spring loaded to accommodate shaft deflections or movement during bearing tolerances, including vertical misalignment due to manufacturing tolerances. The main benefits of using a new generation mechanical seal are, for example, reducing leakage, minimising the possibility of damage to the pump shaft or sleeve, ensuring self-regulation of disc wear, limiting bearing contamination, but also protecting other devices against corrosion and ensuring sealing in a vacuum [33]. An example of identified porosity on a mechanical seal is shown in Figure 2.

The mechanical seal was made of Alloy 410, which is a martensitic stainless steel that is often used in a hardened condition. It is used to provide high strength and moderate resistance to heat and corrosion. The properties of alloy 410 are presented in Table 2, Table 3, Table 4 and Table 5.

The choice of the mechanical seal made of alloy 410 and the porosity cluster resulted from the frequency of identifying this problem in one of the Polish production companies. This non-compliance was identified as part of the non-destructive testing (NDT) using the fluorescence method, which was presented in the studies [71].

After selecting the subject of research, the purpose of the research was defined. This was done by an expert (entity) using the SMART(-ER) method. The purpose of the study was to determine the potential causes of porosity clusters on the 410 alloy mechanical seal and then to analyse them in a sequential and consistent manner to determine the root causes of this non-compliance. The main causes are those whose occurrence has the following simultaneously: (a) the greatest impact causing the formation of porosity clusters and (b) the greatest negative impact on the natural environment. As a result, it will be possible to take improvement actions that address the main causes of the problem. At the same time, these activities will ensure the improvement of product quality and the reduction of the negative impact of its production process on the natural environment.

*Step* *2.* *Selection of a team of experts*

As part of the research, a team of experts was selected, which was responsible for carrying out the process of improving the quality of materials and industrial products. This was done in accordance with the adopted methodology. Initially, the entity applying the method (an expert) selected three experts, mainly paying attention to their knowledge and experience (competence to analyse the selected research problem). They were the NDT research manager, the employee producing the mechanical seal, and the author of the article. Each of these experts indicated two more experts in their group. Due to the fact that the number of experts was the same, Equation (2) was used to estimate the expected number of experts. The result of the calculations is presented by Equation (10):

(10) $$N=\frac{2^{2}\left(2-1\right)}{\left(2^{2}-4\right)+1}=4,$$

It was estimated that at least four experts on the team would be enough to properly analyse the porosity cluster problem on the 410 alloy mechanical seal. Subsequently, the competence factor $K_{k}$ of these experts was calculated using Equations (3) and (4). The degree of knowledge of the problem by the expert (k_z_) and the coefficient of argumentation (k_a_) were determined independently by these experts according to the assessments in Table 1. The result is presented in Equation (11):

(11) $$\begin{array}{cc} K_{k4}=\frac{(8\times 0.1)+\left(0.20+0.15+0.10\right)}{2}=0.62 & K_{k5}=\frac{(7\times 0.1)+\left(0.20+0.17+0.14\right)}{2}=0.61\\ K_{k1}=\frac{(9\times 0.1)+\left(0.50+0.35+0.20\right)}{2}=0.97 & K_{k2}=\frac{(8\times 0.1)+\left(0.50+0.35+0.20\right)}{2}=0.92 \end{array}$$

According to the assumptions, the threshold value of the competence coefficient ($K_{k}$) is 0.6. For none of the experts, this coefficient was lower than this value. Therefore, the selected experts were considered competent to analyse the porosity cluster problem in the mechanical seal of the 410 alloy. The expert team consisted of the NDT research manager (who identified the porosity cluster), the employee producing the mechanical seal and the authors of the article.

*Step* *3.* *Identification of the source of non-compliance*

The team of experts determined the origin (location) of the porosity cluster in the mechanical seal of the 410 alloy. They used brainstorming to do this. It was concluded that the porosity cluster is conditioned by the reaction of gases that dissolve in the liquid metal with the components in the liquid. When the temperature of the metal is lowered, fragments of dissolved gases are separated from the solution. Therefore, it is possible to include these gases in the solidification time. It should be taken into account that dissolved oxides react relatively frequently with carbon. Then, undissolved bubbles are formed (both in the liquid and the solidified metal). Therefore, the team of experts indicated that the source of the porosity cluster on the mechanical seal was the evolution of gases from the metal during its solidification.

*Step* *4.* *Identification of causes of potential non-compliance*

The team of experts identified the potential causes of the porosity cluster on the mechanical seal, i.e., all the causes that were likely to have contributed to this nonconformity. For this purpose, a brainstorming session was carried out by asking the question “What happened that the inconsistency occurred?”. Then, unrealistic causes were removed from all identified causes, i.e., those that practically had no impact on the occurrence of noncompliances, where they were:

- employee stress;
- haste and distraction of the employee;
- dirty form;
- employee errors;
- water in the moulding sand.

For all viable reasons, a cumulative list of potential causes of porosity clustering on the 410 alloy mechanical seal was compiled. The list included the following causes:

- C1—significant nitrogen or hydrogen content in the arc area;
- C2—too high clotting rate;
- C3—formation of reaction metallurgical reactions (reaction in gaseous form);
- C4—inadequate casting design, which causes solidification errors;
- C5—the interaction of iron oxide with carbon, which results in the release of carbon monoxide and carbon dioxide caused by the interaction;
- C6—formation of moisture in the flux (in the case of automatic welding);
- C7—rust on the wire;
- C8—inadequate gas shield;
- C9—electrode humidity;
- C10—small length of service (experience) of the employee;
- C11—occasional periodic training;
- C12—impurities in the moulding sand.

These reasons were further analysed in subsequent stages of the procedure.

*Step* *5.* *Analysis of the causes of potential non-compliance in terms of quality and environment*

The entity (expert) decided that the proposed procedure would be used to simultaneously analyse the impact of potential causes on the occurrence of nonconformities and on the natural environment. On the basis of the qualitative and environmental analyses, the main causes will be identified, and then the proposed improvement actions will be taken for them. Therefore, the entire procedure was carried out, as presented in the next part of the article.

Step 5.1. Determining the degree of influence of potential causes on the occurrence of non-compliance

The degree of mutual influence of potential causes on the formation of a porosity cluster on the mechanical seal was determined. This involved determining how much impact a potential cause has compared to another due to the occurrence of non-compliance. According to the adopted methodology, the degree of impact is determined by dividing points between all potential causes on the list. The higher the number of points, the lesser the cause of the discrepancy. To do this, a decision matrix was developed for pairwise comparisons. The degree of impact of potential causes determining the impact of these causes on the occurrence of noncompliances was compared in pairs. Additionally, the cumulative impact of mutual relationships among potential causes was calculated. The result is presented in Table A1 (Appendix A).

The points awarded reflect the degree of impact of the individual potential causes that determine their impact on the quality of the mechanical seal. On the basis of the cumulative impact, it was possible to determine that the greatest cumulative mutual influence on the formation of the porosity cluster has the cause C2 (value 220); according to ACO, the analysis of the mutual significance of potential causes should start from this with cause. Therefore, this reason was placed first in the matrix (Table 6).

The correct determination of the degree of influence of potential causes supports a consistent and more precise assessment of the correct selection of root causes (those that have the greatest impact on the formation of a porosity cluster in the mechanical seal).

Step 5.2. Determining the degree of environmental impact of the causes of potential non-compliance

Later, the degree of environmental impact for potential causes of porosity clusters in mechanical seals was determined. The idea was to take steps to improve this product in the direction of sustainable production development, i.e., so as to effectively take into account, above all, environmental aspects, i.e., the relationship between the causes of non-compliance and the negative impact of their occurrence on the environment. According to the adopted methodology, the degree of impact is determined by dividing points between all potential causes on the list. The higher the number of points, the less impact the cause has on the environment. The points were recorded in a pairwise comparison matrix. The degree of impact of potential causes on the natural environment was compared in pairs. The result is presented in Table A2 (Appendix A).

For the purposes of these studies, the subject (expert) assumed that the importance of quality to the environment is 1:0.2. Therefore, it was necessary to multiply the values in Table A2 by their weight of 20%, that is, 0.2. Additionally, to use the ACO correctly, it was necessary to calculate the total impact of these causes to determine the cause that has the greatest impact on the natural environment. The result is presented in Table 7.

Based on cumulative mutual influence, it was determined that the greatest influence on the formation of the porosity cluster is caused by the C5 cause (value 41.60). According to the ACO, the analysis of the mutual significance of potential causes should start with this cause. Therefore, this reason was placed first in the matrix (Table A3 (Appendix A)).

The assigned values are the degree of impact of the individual potential causes and determine their impact on the natural environment.

Step 5.3. Determination of the simultaneous impact of the causes of potential non-compliance in terms of quality and environment

To carry out a comprehensive analysis of the qualitative and environmental impact of the causes of potential noncompliances, it is necessary to combine the matrix developed in step 5.1 with the matrix developed in step 5.2. Therefore, a third decision matrix was created that contained the sum of the values of these matrices. Furthermore, the total mutual qualitative and environmental impact of these causes was calculated, as shown in Table A4 (Appendix A).

It was observed that the ACO analysis should start with the potential cause C2, due to the fact that it had the greatest cumulative impact. Hence, the matrix was ordered so that this cause was first in the rows and columns of this matrix (Table A5 (Appendix A)).

Based on the values of this matrix, the next step of the procedure was implemented, as presented in the next part of the article.

Step 5.4. Analysis of the mutual significance of potential causes of noncompliance in terms of quality and environment

Three developed decision matrices (from earlier steps of the procedure) were used to determine the root causes of noncompliance due to their impact on the occurrence of noncompliance and on the natural environment. It was a matrix: qualitative (Table 6), environmental (Table A3), and quality-environmental (Table A5).

In this case, it is the determination of the queue (ranking) of potential causes, both in terms of their impact on the occurrence of non-conformities and their impact on the natural environment. As assumed, ant colony optimisation (ACO) was used for this purpose, which was used three times by introducing values from three developed decision matrices. To efficiently and dynamically search for the main causes of porosity clusters according to ACO, the MATLAB programme was used. MATLAB software implements an algorithm developed based on the example presented in [60,68].

**Figure 3:**
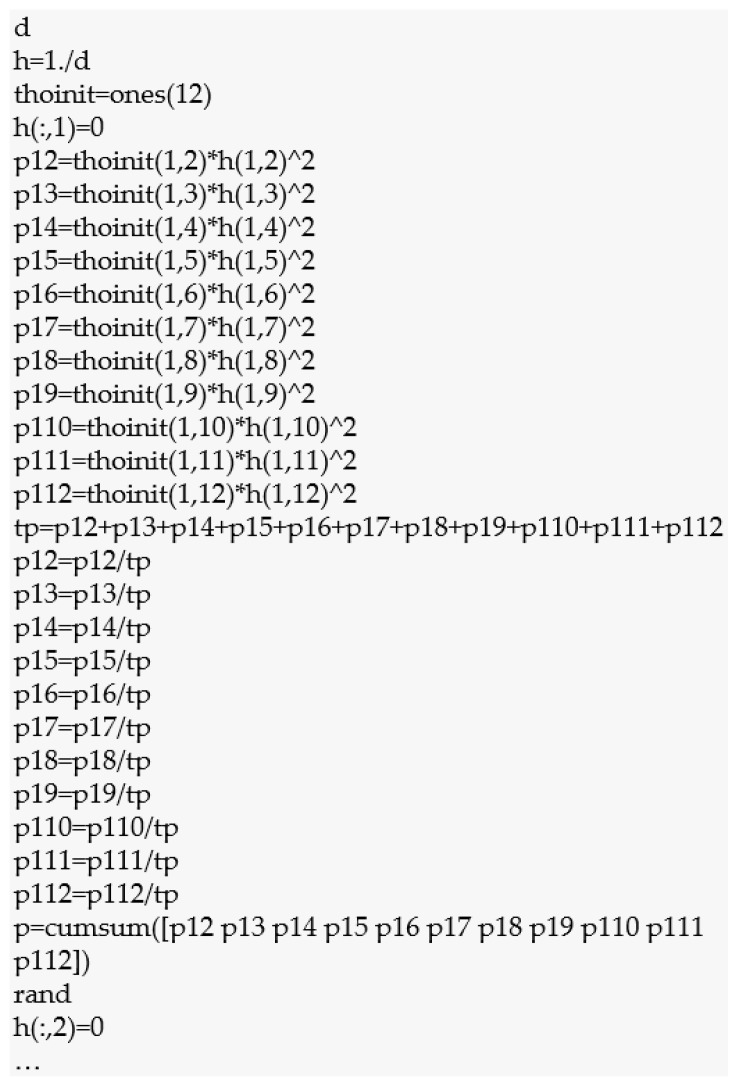
Fragment of the ACO algorithm initiated in the MATLAB programmw for the degree of influence of the causes of the porosity cluster on the mechanical seal.

Initially, the degree of impact of the causes of non-compliance was subjected to the ACO analysis, where this degree determined the impact of these causes on the formation of a porosity cluster on the mechanical seal made of alloy 410. A fragment of the algorithm supporting the calculation of the ACO for the degree of impact of the causes of non-conformity is shown in Figure 3.

Initially, a decision matrix (d) was initialised that contained the non-conformity causes of the degree of influence of the porosity cluster on the mechanical seal 410. Subsequently, the visibility (h) between the individual non-conforming causes was defined. According to Formula (6), this means determining the inverse of the value of the decision matrix for potential causes, which concerns the calculation of ηij=1dij—the heuristic value of the shift from point i to point j. The result is presented in Table 8.

Subsequently, as in Equation (6), the same number of pheromones $\tau_{ij}=1$ was assumed for each potential cause. Determining the significance of the impact of potential causes on the formation of porosity clusters started with the conventionally marked cause C1. Therefore, all values for the cause column C1 were 0, and the rest remained unchanged. Then, using Equation (7), the probability with which the first ant k will decide to move from the first cause (C1) to the next cause $j\in N_{i}^{k}$ was calculated in order to create a path in the iteration of the algorithm, where α = 1 and β = 2. To do this, the probability of the distance from the degree of influence of the potential cause C1 (significant nitrogen or hydrogen content in the area of the arc) to the degree of influence of all other potential causes (12) was calculated:

(12) $$\begin{array}{cccc} \tau_{12}{\left[\eta_{12}\right]}^{2}=0.0025 & \tau_{15}{\left[\eta_{15}\right]}^{2}=0.0059 & \tau_{18}{\left[\eta_{18}\right]}^{2}=0.0017 & \tau_{111}{\left[\eta_{111}\right]}^{2}=0.0010\\ \tau_{13}{\left[\eta_{13}\right]}^{2}=0.0031 & \tau_{16}{\left[\eta_{16}\right]}^{2}=0.1111 & \tau_{19}{\left[\eta_{19}\right]}^{2}=0.0014 & \tau_{112}{\left[\eta_{112}\right]}^{2}=0.0019\\ \tau_{14}{\left[\eta_{14}\right]}^{2}=0.0278 & \tau_{17}{\left[\eta_{17}\right]}^{2}=0.0015 & \tau_{110}{\left[\eta_{110}\right]}^{2}=0.0012 & \end{array}$$

The total value of the probability of travelling the distance from the value of the degree of influence of the first potential cause to the values of the degree of influence of all other potential causes was 0.1591. Subsequently, according to Equation (7), the probability that the first ant passed from the degree of influence of potential causes first through all other degrees of influence of potential causes was calculated. The results are presented in Equation (13):

(13) $$\begin{array}{cccc} p_{12}^{1}=\frac{0.0025}{0.1591}=0.0157 & p_{15}^{1}=\frac{0.0059}{0.1591}=0.0372 & p_{18}^{1}=\frac{0.0017}{0.1591}=0.0109 & p_{111}^{1}=\frac{0.0010}{0.1591}=0.0065\\ p_{13}^{1}=\frac{0.0031}{0.1591}=0.0194 & p_{16}^{1}=\frac{0.1111}{0.1591}=0.0066 & p_{19}^{1}=\frac{0.0014}{0.1591}=0.0086 & p_{112}^{1}=\frac{0.0019}{0.1591}=0.0119\\ p_{14}^{1}=\frac{0.0278}{0.1591}=0.1746 & p_{17}^{1}=\frac{0.0015}{0.1591}=0.0093 & p_{110}^{1}=\frac{0.0012}{0.1591}=0.0075 & \end{array}$$

Later, using the Equations (8) and (9), the cumulative value ${cp}_{ij}^{k}$ was calculated for the value of the transition probability from the degree of influence of the first potential cause to the degree of influence of other potential causes of the porosity cluster (14):

(14) $$\begin{array}{cc} cp_{12}^{1}=0.0157 & cp_{18}^{1}=0.9546+0.0109=0.9655\\ \begin{array}{c} cp_{13}^{1}=0.0157+0.0194=0.0351\\ \begin{array}{c} cp_{14}^{1}=0.0351+0.1746=0.2097\\ \begin{array}{c} cp_{15}^{1}=0.2097+0.0372=0.2469\\ \begin{array}{c} cp_{16}^{1}=0.2469+0.0066=0.9453\\ cp_{17}^{1}=0.9453+0.0093=0.9546 \end{array} \end{array} \end{array} \end{array} & \begin{array}{c} cp_{19}^{1}=0.9655+0.0086=0.9741\\ \begin{array}{c} cp_{110}^{1}=0.9741+0.0075=0.9816\\ \begin{array}{c} \begin{array}{cc} cp_{111}^{1}=0.9816+0.0065=0.9881 & \end{array}\\ \begin{array}{c} cp_{112}^{1}=0.9881+0.0119=1.0000 \end{array} \end{array} \end{array} \end{array} \end{array}$$

Later, following the authors of articles [60,68] a random number was randomly drawn (with the use of MATLAB software) and amounted to 0.0950.

This number was more comparable to (but not greater than) the fourth cumulative value ${cp}_{4}^{1}$, where 0.0950≅0.2097. Therefore, from the first potential cause, the ant goes first to the fourth potential cause. Again, all values in the four-part potential cause column were reset to zero, and the other values were left unchanged. Then, the calculations were carried out adequately for the potential cause and were repeated accordingly for the subsequent potential causes of the porosity cluster on the mechanical seal. Later, an algorithm was initiated in MATLAB, an example of which is presented in [60,68]. It was assumed that the number of iterations (the number of passages of ants) was equal to 100. However, the number of ants was equal to 50. After the ants had covered all the paths, the pheromone on all edges could evaporate. Afterward, each ant k leaves the pheromone $∆\tau_{ij}^{k}\left(t\right)$ on the edges it has visited, as shown by patterns (8) and (9). As a result, a ranking of the potential causes of the porosity cluster in the 410 steel mechanical seal was determined, where the aim was to arrange the potential causes from the most to the least contributing to the non-conformity (15):

The best tour = C1, C6, C4, C5, C2, C3, C12, C8, C7, C9, C10, C11, C1(15)

where: C1—too high clotting rate, C2—significant nitrogen or hydrogen content in the arc area; C3–C12 as in the causes list.

The sum of the degrees of mutual influence of such ordered potential causes was 66, as shown in Table 9.

The most favourable order of ranking potential causes of the porosity cluster is as follows: too high solidification rate; formation of moisture in the flux (in the case of automatic welding); inadequate casting design, which determines solidification errors; interaction of iron oxide with carbon, which causes the release of carbon monoxide and carbon dioxide due to impact; etc.

Subsequently, the ACO procedure was repeated, but in order to determine the root causes of non-conformities due to their impact on the natural environment. MATLAB software was used for this. ACO analysis was carried out for the values of the pairwise comparison matrix, which referred to the degree of negative impact of the causes of the porosity cluster on the natural environment. As a result, a ranking of the potential causes of the porosity cluster on the 410 steel mechanical seal was determined, where the aim was to arrange the potential causes from the most to the least affecting the environment (16):

The best tour = C1, C2, C4, C6, C12, C7, C3, C8, C5, C9, C10, C11, C1(16)

where: C1—interaction of iron oxide with carbon, which causes the release of carbon monoxide and carbon dioxide due to the interaction, C2—significant nitrogen or hydrogen content in the arc area, C3—solidification rate too high; C4—formation of metallurgical reactions (reaction in gaseous form); C5—inadequate casting design, which determines solidification errors; and C6–C12 as in the list of causes.

The classification of potential causes arranged in this way ensures that the sum of the degrees of mutual influence of these causes on the environment is 12.40, as in Table 10.

The most favourable order of ranking potential causes of porosity clusters due to their impact on the natural environment is as follows: interaction of iron oxide with carbon, which causes the release of carbon monoxide and carbon dioxide caused by the interaction; significant nitrogen or hydrogen content in the arc area, after the state of reaction metallurgical reactions (reaction in a gaseous form); unsuitable construction of the casting, which determines solidification errors; etc.

Subsequently, ACO was carried out on the basis of a decision matrix developed for the simultaneous impact of potential causes on the occurrence of nonconformities (in terms of quality) and on the natural environment. The data for analysis are presented in Table 11, i.e., data from a matrix containing the values of the simultaneous impact of the causes of potential noncompliance on the quality of the material or product and on the natural environment. The ACO procedure was repeated using MATLAB software. As a result, a ranking of the potential causes of the porosity group was determined, the objective of which was to arrange the potential causes from the most to the least affecting both the deterioration of the quality of the mechanical seal and the environment (17):

The best tour = C1, C6, C4, C5, C3, C2, C12, C8, C7, C9, C10, C11, C1(17)

where: C1—too high solidification rate, C2—significant nitrogen or hydrogen content in the arc area; and C3–C12 as in the list of causes.

The sum of the degrees of mutual influence of such ordered potential causes was 79.5, as shown in Table 11.

After conducting a qualitative and environmental analysis of the potential causes of the porosity cluster on the mechanical seal made of alloy 410, it was possible to identify the main causes. This represents the next stage of the procedure.

*Step* *6.* *Identification of the causes of the main non-conformities and proposing improvement actions*

The purpose of this stage was to identify the main causes of porosity concentration in the mechanical seal of the 410 alloy. In the proposed approach, the expert (entity) using the procedure decided to conduct a comprehensive quality and environmental analysis, after which a ranking of potential causes was obtained, determining the simultaneous impact of potential causes on the formation of compliance and the environment. It was created after the ACO method, where the sequence of causes is presented by Formula (16). Given the ranking of the causes of the cluster of porosities, it was possible to determine the main causes of this problem. As assumed, the Pareto principle (20/80) was used for this purpose. The result is presented in Table 12.

According to this analysis, the main reasons for the porosity cluster in the mechanical seal were: a too high solidification rate, the formation of moisture in the flux (in the case of automatic welding), and inadequate casting design, which determines solidification errors. For these reasons, improvements should be taken first. Then it is possible to significantly improve the quality of the mechanical seal and eliminate its negative impact on the natural environment. The final decision on possible improvement actions belongs to the entity (expert, broker) and may depend on the production and financial capabilities, or the possibility of correlating various modifications of the criteria states with each other.

## 4. Discussion

Improving the quality of materials and industrial products relatively often concerns the first phase of the improvement process, i.e., the identification of non-conformities arising from them [48,72,73]. However, it is still a challenge to take actions that allow dynamic and precise analysis of the causes of noncompliance in terms of quality and environment [74]. To identify the main causes of these noncompliances as the causes that have the greatest impact on the deterioration of the quality of materials and production processes, as well as the greatest negative impact on the natural environment [75,76]. The idea behind such a procedure is to determine in a proper manner the order of elimination or reduction of the causes of non-conformities through the selection of adequate improvement actions. Previous research has not presented such an approach in a coherent and uniform manner, including when supported by machine learning algorithms combined with other techniques. In general, the focus was on the use of traditional quality management tools, e.g., [29,31,32,33]. There have also been articles using hybrid quality management tools and multi-criteria decision support methods [34,35]. However, no procedure has been found that would support the process of analysing the causes of noncompliances identified on any materials or industrial products, where these analyses come down to identifying the main causes of these non-compliances, which will be responsible for both the deterioration of quality and the natural environment.

Therefore, the objective of the investigation was to develop a procedure to identify critical causes of material incompatibility. The procedure was tested for a new-generation mechanical seal made of alloy 410. The fluorescence method (non-destructive testing) relatively often detected a common noncompliance, i.e., a cluster of porosity. After the procedure test, rankings of the reasons for the formation of porosity clusters on the mechanical seal were obtained, which concerned the impact of these causes on the deterioration of the product quality as well as the negative impact of these causes on the natural environment. The rankings obtained are presented in Table 13.

A relatively large convergence of the sequence of potential causes in the qualitative and quality-environmental rankings was demonstrated. It was also observed that the environmental ranking differs significantly from the qualitative and quality-environmental rankings. Therefore, it was concluded that in this case, the impact on the final quality-environmental classification resulted from the adopted ratio of quality to environment (1.0:0.2). At the same time, it is possible to observe that the proposed procedure is effective in this type of analysis, but the environment should be presented to a greater extent. This procedure can be used to analyse the causes of potential non-compliance only in terms of the impact on the quality of materials and products, only in terms of the impact on the natural environment, or simultaneously in terms of the quality and environmental impact. The type of analysis results from the needs of the entity using the proposed procedure. At the same time, the procedure test allowed to confirm the adopted hypothesis that the pro-quality improvement of materials and industrial products can be made on the basis of rankings of the causes of non-compliance of these materials and products, where these rankings will be created after a coherent and sequential analysis of the mutual degree of the impact of these causes on: (a) the occurrence of non-compliance (deterioration of the quality of materials and products), (b) and/or on the natural environment (negative impact on the environment of the causes of potential non-compliance of materials and products). Hypothesis 1 is verified.

Therefore, the main benefits of the proposed procedure can be identified, i.e.:

- improving the quality of materials or products according to a precisely defined ranking of the causes of non-compliance arising from them;
- reducing the negative impact of the causes of non-compliance arising in materials or products by taking appropriate improvement actions;
- improving the quality of materials or processes while ensuring the reduction of the negative impact of their creation;
- setting a sequence of improvement actions that can be undertaken in three aspects: improving quality, reducing the negative impact on the natural environment, and simultaneously improving quality with a view to caring for the natural environment;
- streamlining the pro-quality process of making decisions on activities that improve the materials and products of their creation;
- reduction of the subjectivity of the team of experts by supporting the decision-making process with computer software.

However, the limitations of the proposed procedure are, e.g., the need to select a team of experts among people competent and familiar with the problem, as well as the time-consuming process of analysis in the case of a large number of potential causes. Additionally, this procedure does not guarantee that each cause will be included in the analysis. However, it is highly likely that causes with a higher probability of affecting the problem will be included (i.e., those that may have a significant impact on the problem). It results from the assumption that a team of experts has knowledge about the problem. Hence, if experts are competent people, the degree of probability that important causes will not be omitted is higher.

Further analyses will consist of verifying the procedure on other materials or industrial products. In addition, it is planned to analyse the differences caused by the selection of a different quality-environment relationship, so as to reduce the methodology to other, more general assumptions of the procedure.

## 5. Conclusions

Stabilisation of the quality of materials and products is supported by quality tests. This difficult task becomes even more difficult in the face of the need to apply the principles of sustainable development to competitive enterprises. In this case, solutions should be sought to ensure both high quality and minimise the negative impact on the natural environment. The aim of the investigation was to develop a procedure to identify critical causes of material incompatibility. The procedure was tested for a new-generation mechanical seal made of alloy 410. In this product, a discrepancy was identified relatively frequently, which was a cluster of porosity. As a result, twelve potential causes were identified, i.e., those that probably affect the occurrence of noncompliance. These reasons were further analysed, e.g., in pairwise comparison decision matrices and then according to ACO (ant colony optimisation), which was supported by the MATLAB programme. Using the Pareto-Lorenz method, the main reasons why improvement activities should be initiated in the first place were selected in order to significantly improve the quality of the product and mitigate the negative impact on the natural environment. The main reasons were: a too high rate of solidification, the formation of moisture in the flux, and the inadequate design of the casting.

The novelty of the procedure is the precise and dynamic identification of a queue of improvement actions that will be undertaken only for the most important causes of non-compliance with materials and industrial products. The originality of the procedure is to support the process of analysing the causes of non-conformities identified in any materials or industrial products, where these analyses come down to identifying the main causes of these non-conformities, which will be responsible for both the deterioration of quality and the natural environment.

After making adequate assumptions, the procedure can be used to analyse any causes of non-compliance that have been detected by any quality controls, including for materials and industrial products.

## Figures and Tables

**Table 1:** Values to determine the expert’s competence factor. Own study based on [33].

Value of Points for Expert Self-Assessment				
Points	Description			
0–1	The expert does not know the problem			
2–3	The expert knows the problem poorly, but it is within his area of interest			
4–6	The expert knows the problem satisfactorily, but has no part in its practical solution			
7–9	The expert knows the problem well and participates in its practical solution			
10	The expert knows the problem perfectly well, where this problem belongs to the narrow specialization of the expert			
**Argumentation Coefficient k_a_**				
Root		Argumentation		
		$a_{1}$	$a_{2}$	$a_{3}$
Theoretical analysis conducted by an expert		0.20	0.15	0.10
Practical experience of an expert		0.50	0.35	0.20
Knowledge of the works of native authors		0.05	0.04	0.03
Knowledge of works by foreign authors		0.05	0.04	0.03
Expert intuition		0.20	0.17	0.14

**Table 2:** Alloy 410 properties—mechanical and physical. Own study based on [33,58,70].

Mechanical and Physical Properties	21 °C	100 °C	500 °C	649 °C	788 °C
Thermal expansion coefficient [μm/m°C]	–	9.8	11.2	11.7	11.9
Thermal conductivity [kcal/°C]	–	21.4	24.7	–	–
Coefficient of elasticity [$\times 10^{5}\;MPa$]	2	–	–	–	–

**Table 3:** Properties of alloy 410—tensile strength [16].

Tensile Strength, ksi	60–75
0.2% yield strength [ksi]	32–42
Elongation [%]	20–40
Surface reduction [%]	50–75

**Table 4:** Properties of alloy 410—tempering temperature. Own study based on [33,58,70].

Tempering Temperature [°C]	–	149	260	371	566	621	649	704	760	816
Tensile strength [ksi]	193.5	188.5	181.6	181.4	124.1	117.5	113	101.8	96.5	131.8
0.2% yield strength	149.8	148.6	143.6	144.7	110.3	103.7	99.1	84.2	77.9	88.6
Elongation, %	17	17.3	16.8	16	20.8	21.3	22	23.5	25	19.5
Surface reduction, %	56.8	59.7	61.1	61.1	67.2	66.1	66.5	68.8	69.9	59.6
Brinell hardness	388	388	361	361	255	235	229	207	189	257

**Table 5:** Chemical composition of alloy 410. Own study based on [33,58,70].

[%]	Cr	Mn	Ni	C	Si	P	S	Fe
Min.	11.5	–	–	0.08	–	–	–	–
Maks.	13.5	1	0.75	0.15	1	0.04	0.03	Balance

**Table 6:** An ordered matrix of pairwise comparisons for the degree of influence of potential causes determining the impact of these causes on the formation of a porosity cluster on a mechanical seal made of alloy 410.

	C2	C1	C3	C4	C5	C6	C7	C8	C9	C10	C11	C12	Total Impact
C2	0	20	18	6	13	3	26	24	27	29	31	23	220
C1	20	0	2	14	7	17	6	4	7	9	11	3	100
C3	18	2	0	12	5	15	8	6	9	11	13	5	104
C4	6	14	12	0	7	3	20	18	21	23	25	17	166
C5	13	7	5	7	0	10	13	11	14	16	18	10	124
C6	3	17	15	3	10	0	23	21	24	26	28	20	190
C7	26	6	8	20	13	23	0	2	1	3	5	3	110
C8	24	4	6	18	11	21	2	0	3	5	7	1	102
C9	27	7	9	21	14	24	1	3	0	2	4	4	116
C10	29	9	11	23	16	26	3	5	2	0	2	6	132
C11	31	11	13	25	18	28	5	7	4	2	0	8	152
C12	23	3	5	17	10	20	3	1	4	6	8	0	100

where: C1–C12 as in the list of causes.

**Table 7:** Pairwise comparison matrix taking into account the importance of the value of the degree of negative impact of potential causes on the natural environment for the problem of porosity cluster on a mechanical seal made of alloy 410.

	C1	C2	C3	C4	C5	C6	C7	C8	C9	C10	C11	C12	Total Impact
C1	0.00	2.80	0.40	4.00	1.00	1.40	2.40	3.20	4.40	4.80	5.20	2.00	31.60
C2	2.80	0.00	2.40	1.20	3.80	1.40	0.40	0.40	1.60	2.00	2.40	0.80	19.20
C3	0.40	2.40	0.00	3.60	1.40	1.00	2.00	2.80	4.00	4.40	4.80	1.60	28.40
C4	4.00	1.20	3.60	0.00	5.00	2.60	1.60	0.80	0.40	0.80	1.20	2.00	23.20
C5	1.00	3.80	1.40	5.00	0.00	2.40	3.40	4.20	5.40	5.80	6.20	3.00	41.60
C6	1.40	1.40	1.00	2.60	2.40	0.00	1.00	1.80	3.00	3.40	3.80	0.60	22.40
C7	2.40	0.40	2.00	1.60	3.40	1.00	0.00	0.80	2.00	2.40	2.80	0.40	19.20
C8	3.20	0.40	2.80	0.80	4.20	1.80	0.80	0.00	1.20	1.60	2.00	1.20	20.00
C9	4.40	1.60	4.00	0.40	5.40	3.00	2.00	1.20	0.00	0.40	0.80	2.40	25.60
C10	4.80	2.00	4.40	0.80	5.80	3.40	2.40	1.60	0.40	0.00	0.40	2.80	28.80
C11	5.20	2.40	4.80	1.20	6.20	3.80	2.80	2.00	0.80	0.40	0.00	3.20	32.80
C12	2.00	0.80	1.60	2.00	3.00	0.60	0.40	1.20	2.40	2.80	3.20	0.00	20.00

where: C1–C12 as in the causes list.

**Table 8:** Values for the visibility of the distance between potential causes of porosity clusters on a 410 alloy mechanical seal.

	C2	C1	C3	C4	C5	C6	C7	C8	C9	C10	C11	C12
C2	0.00	0.05	0.06	0.17	0.08	0.33	0.04	0.04	0.04	0.03	0.03	0.04
C1	0.05	0.00	0.50	0.07	0.14	0.06	0.17	0.25	0.14	0.11	0.09	0.33
C3	0.06	0.50	0.00	0.08	0.20	0.07	0.13	0.17	0.11	0.09	0.08	0.20
C4	0.17	0.07	0.08	0.00	0.14	0.33	0.05	0.06	0.05	0.04	0.04	0.06
C5	0.08	0.14	0.20	0.14	0.00	0.10	0.08	0.09	0.07	0.06	0.06	0.10
C6	0.33	0.06	0.07	0.33	0.10	0.00	0.04	0.05	0.04	0.04	0.04	0.05
C7	0.04	0.17	0.13	0.05	0.08	0.04	0.00	0.50	1.00	0.33	0.20	0.33
C8	0.04	0.25	0.17	0.06	0.09	0.05	0.50	0.00	0.33	0.20	0.14	1.00
C9	0.04	0.14	0.11	0.05	0.07	0.04	1.00	0.33	0.00	0.50	0.25	0.25
C10	0.03	0.11	0.09	0.04	0.06	0.04	0.33	0.20	0.50	0.00	0.50	0.17
C11	0.03	0.09	0.08	0.04	0.06	0.04	0.20	0.14	0.25	0.50	0.00	0.13
C12	0.04	0.33	0.20	0.06	0.10	0.05	0.33	1.00	0.25	0.17	0.13	0.00

where: C1–C12 as in the causes list.

**Table 9:** ACO result, including ranking of potential causes in terms of their impact on the formation of a porosity cluster on the mechanical seal of alloy 410.

	C2	C1	C3	C4	C5	C6	C7	C8	C9	C10	C11	C12
C2	0	20	2	14	7	17	6	4	7	9	11	3
C1	20	0	18	6	13	3	26	24	27	29	31	23
C3	2	18	0	12	5	15	8	6	9	11	13	5
C4	14	6	12	0	7	3	20	18	21	23	25	17
C5	7	13	5	7	0	10	13	11	14	16	18	10
C6	17	3	15	3	10	0	23	21	24	26	28	20
C7	6	26	8	20	13	23	0	2	1	3	5	3
C8	4	24	6	18	11	21	2	0	3	5	7	1
C9	7	27	9	21	14	24	1	3	0	2	4	4
C10	9	29	11	23	16	26	3	5	2	0	2	6
C11	11	31	13	25	18	28	5	7	4	2	0	8
C12	3	23	5	17	10	20	3	1	4	6	8	0

where: C1–C12 as in the list of causes.

**Table 10:** ACO results rank the potential causes in terms of their environmental impact for the porosity cluster problem on the 410 alloy mechanical seal.

	C5	C1	C2	C3	C4	C6	C7	C8	C9	C10	C11	C12
C5	0.00	1.00	3.80	1.40	5.00	2.40	3.40	4.20	5.40	5.80	6.20	3.00
C1	1.00	0.00	2.80	0.40	4.00	1.40	2.40	3.20	4.40	4.80	5.20	2.00
C2	3.80	2.80	0.00	2.40	1.20	1.40	0.40	0.40	1.60	2.00	2.40	0.80
C3	1.40	0.40	2.40	0.00	3.60	1.00	2.00	2.80	4.00	4.40	4.80	1.60
C4	5.00	4.00	1.20	3.60	0.00	2.60	1.60	0.80	0.40	0.80	1.20	2.00
C6	2.40	1.40	1.40	1.00	2.60	0.00	1.00	1.80	3.00	3.40	3.80	0.60
C7	3.40	2.40	0.40	2.00	1.60	1.00	0.00	0.80	2.00	2.40	2.80	0.40
C8	4.20	3.20	0.40	2.80	0.80	1.80	0.80	0.00	1.20	1.60	2.00	1.20
C9	5.40	4.40	1.60	4.00	0.40	3.00	2.00	1.20	0.00	0.40	0.80	2.40
C10	5.80	4.80	2.00	4.40	0.80	3.40	2.40	1.60	0.40	0.00	0.40	2.80
C11	6.20	5.20	2.40	4.80	1.20	3.80	2.80	2.00	0.80	0.40	0.00	3.20
C12	3.00	2.00	0.80	1.60	2.00	0.60	0.40	1.20	2.40	2.80	3.20	0.00

where: C1—interaction of iron oxide with carbon, which causes the release of carbon monoxide and carbon dioxide due to the interaction, C2—significant nitrogen or hydrogen content in the arc area, C3—solidification rate too high; C4—metallurgical reaction (reaction in gaseous form); C5—improper casting design, which determines solidification errors; C6–C12 as in the list of causes.

**Table 11:** ACO result taking into account the ranking of potential causes in terms of their impact on the formation of a porosity cluster on the mechanical seal made of alloy 410 and, at the same time, their impact on the environment.

	C2	C1	C3	C4	C5	C6	C7	C8	C9	C10	C11	C12
C2	0.00	22.80	20.40	7.20	16.80	4.40	26.40	24.40	28.60	31.00	33.40	23.80
C1	22.80	0.00	2.40	18.00	8.00	18.40	8.40	7.20	11.40	13.80	16.20	5.00
C3	20.40	2.40	0.00	15.60	6.40	16.00	10.00	8.80	13.00	15.40	17.80	6.60
C4	7.20	18.00	15.60	0.00	12.00	5.60	21.60	18.80	21.40	23.80	26.20	19.00
C5	34.00	0.00	4.00	34.00	12.00	24.00	18.00	20.00	29.00	33.00	37.00	13.00
C6	4.40	18.40	16.00	5.60	12.40	0.00	24.00	22.80	27.00	29.40	31.80	20.60
C7	26.40	8.40	10.00	21.60	16.40	24.00	0.00	2.80	3.00	5.40	7.80	3.40
C8	24.40	7.20	8.80	18.80	15.20	22.80	2.80	0.00	4.20	6.60	9.00	2.20
C9	28.60	11.40	13.00	21.40	19.40	27.00	3.00	4.20	0.00	2.40	4.80	6.40
C10	31.00	13.80	15.40	23.80	21.80	29.40	5.40	6.60	2.40	0.00	2.40	8.80
C11	33.40	16.20	17.80	26.20	24.20	31.80	7.80	9.00	4.80	2.40	0.00	11.20
C12	23.80	5.00	6.60	19.00	13.00	20.60	3.40	2.20	6.40	8.80	11.20	0.00

where: C1–C12 as in the causes list.

**Table 12:** Selection of root causes of porosity cluster on alloy 410 mechanical seal.

No.	Potential Causes	Ranking (According to ACO)	Cause Category
C2	too high clotting rate	1	Main causes (the most important)
C6	formation of moisture in the flux (in case of automatic welding)	2	
C4	inadequate casting design, which causes solidification errors	3	
C5	the interaction of iron oxide with carbon, which results in the release of carbon monoxide and carbon dioxide caused by the interaction	4	Potential causes (less important)
C3	formation of reaction metallurgical reactions (reaction in gaseous form)	5	
C1	significant nitrogen or hydrogen content in the arc area	6	
C12	impurities in the molding sand	7	
C8	inadequate gas shield	8	
C7	rust on the wire	9	
C9	electrode humidity	10	
C10	small length of service (experience) of the employee	11	
C11	occasional periodic training	12	

**Table 13:** Comparative analysis of ACO rankings for the causes of porosity cluster on a 410 alloy mechanical seal.

Sequence of Causes	Qualitative Ranking	Environmental Ranking	Qualitative-Environmental Ranking	Cause Category
1	C2	C5	C2	Main causes (the most important)
2	C6	C1	C6	
3	C4	C3	C4	
4	C5	C6	C5	Potential causes (less important)
5	C1	C12	C3	
6	C3	C7	C1	
7	C12	C2	C12	
8	C8	C8	C8	
9	C7	C4	C7	
10	C9	C9	C9	
11	C10	C10	C10	
12	C11	C11	C11	

## Data Availability

Not applicable.

## Appendix A

**Table A1:** Matrix of pairwise comparisons for the degree of impact of potential causes determining the impact of these causes on the formation of a porosity cluster on a mechanical seal made of alloy 410.

	C1	C2	C3	C4	C5	C6	C7	C8	C9	C10	C11	C12	Total Impact
C1	0	20	2	14	7	17	6	4	7	9	11	3	100
C2	20	0	18	6	13	3	26	24	27	29	31	23	220
C3	2	18	0	12	5	15	8	6	9	11	13	5	104
C4	14	6	12	0	7	3	20	18	21	23	25	17	166
C5	7	13	5	7	0	10	13	11	14	16	18	10	124
C6	17	3	15	3	10	0	23	21	24	26	28	20	190
C7	6	26	8	20	13	23	0	2	1	3	5	3	110
C8	4	24	6	18	11	21	2	0	3	5	7	1	102
C9	7	27	9	21	14	24	1	3	0	2	4	4	116
C10	9	29	11	23	16	26	3	5	2	0	2	6	132
C11	11	31	13	25	18	28	5	7	4	2	0	8	152
C12	3	23	5	17	10	20	3	1	4	6	8	0	100

where: C1–C12 as in the list of causes.

**Table A2:** Pairwise comparison matrix for the value of the degree of negative impact of potential causes on the natural environment for the problem of porosity cluster on a mechanical seal made of alloy 410.

	C1	C2	C3	C4	C5	C6	C7	C8	C9	C10	C11	C12
C1	0	7	4	15	12	6	9	14	9	19	21	8
C2	7	0	3	8	19	1	2	7	2	12	14	1
C3	4	3	0	11	16	2	5	10	5	15	17	4
C4	15	8	11	0	27	9	6	1	6	4	6	7
C5	12	19	16	27	0	18	21	26	21	31	33	20
C6	6	1	2	9	18	0	3	8	3	13	15	2
C7	9	2	5	6	21	3	0	5	0	10	12	1
C8	14	7	10	1	26	8	5	0	5	5	7	6
C9	9	2	5	6	21	3	0	5	0	10	12	1
C10	19	12	15	4	31	13	10	5	10	0	2	11
C11	21	14	17	6	33	15	12	7	12	2	0	13
C12	8	1	4	7	20	2	1	6	1	11	13	0

where: C1–C12 as in the causes list.

**Table A3:** An ordered matrix of pairwise comparisons taking into account the importance of the value of the degree of negative impact of potential causes on the natural environment for the problem of porosity cluster on the mechanical seal made of alloy 410.

	C5	C1	C2	C3	C4	C6	C7	C8	C9	C10	C11	C12	Total Impact
C5	0.00	1.00	3.80	1.40	5.00	2.40	3.40	4.20	5.40	5.80	6.20	3.00	41.60
C1	1.00	0.00	2.80	0.40	4.00	1.40	2.40	3.20	4.40	4.80	5.20	2.00	31.60
C2	3.80	2.80	0.00	2.40	1.20	1.40	0.40	0.40	1.60	2.00	2.40	0.80	19.20
C3	1.40	0.40	2.40	0.00	3.60	1.00	2.00	2.80	4.00	4.40	4.80	1.60	28.40
C4	5.00	4.00	1.20	3.60	0.00	2.60	1.60	0.80	0.40	0.80	1.20	2.00	23.20
C6	2.40	1.40	1.40	1.00	2.60	0.00	1.00	1.80	3.00	3.40	3.80	0.60	22.40
C7	3.40	2.40	0.40	2.00	1.60	1.00	0.00	0.80	2.00	2.40	2.80	0.40	19.20
C8	4.20	3.20	0.40	2.80	0.80	1.80	0.80	0.00	1.20	1.60	2.00	1.20	20.00
C9	5.40	4.40	1.60	4.00	0.40	3.00	2.00	1.20	0.00	0.40	0.80	2.40	25.60
C10	5.80	4.80	2.00	4.40	0.80	3.40	2.40	1.60	0.40	0.00	0.40	2.80	28.80
C11	6.20	5.20	2.40	4.80	1.20	3.80	2.80	2.00	0.80	0.40	0.00	3.20	32.80
C12	3.00	2.00	0.80	1.60	2.00	0.60	0.40	1.20	2.40	2.80	3.20	0.00	20.00

where: C1–C12 as in the causes list.

**Table A4:** Matrix containing the cumulative impact of the interrelations of potential causes in terms of quality and environment for the problem of porosity cluster on the mechanical seal made of alloy 410.

	C1	C2	C3	C4	C5	C6	C7	C8	C9	C10	C11	C12	Total Impact
C1	0.00	22.80	2.40	18.00	8.00	18.40	8.40	7.20	11.40	13.80	16.20	5.00	131.6
C2	22.80	0.00	20.40	7.20	16.80	4.40	26.40	24.40	28.60	31.00	33.40	23.80	239.2
C3	2.40	20.40	0.00	15.60	6.40	16.00	10.00	8.80	13.00	15.40	17.80	6.60	132.4
C4	18.00	7.20	15.60	0.00	12.00	5.60	21.60	18.80	21.40	23.80	26.20	19.00	189.2
C5	8.00	16.80	6.40	12.00	0.00	12.40	16.40	15.20	19.40	21.80	24.20	13.00	165.6
C6	18.40	4.40	16.00	5.60	12.40	0.00	24.00	22.80	27.00	29.40	31.80	20.60	212.4
C7	8.40	26.40	10.00	21.60	16.40	24.00	0.00	2.80	3.00	5.40	7.80	3.40	129.2
C8	7.20	24.40	8.80	18.80	15.20	22.80	2.80	0.00	4.20	6.60	9.00	2.20	122
C9	11.40	28.60	13.00	21.40	19.40	27.00	3.00	4.20	0.00	2.40	4.80	6.40	141.6
C10	13.80	31.00	15.40	23.80	21.80	29.40	5.40	6.60	2.40	0.00	2.40	8.80	160.8
C11	16.20	33.40	17.80	26.20	24.20	31.80	7.80	9.00	4.80	2.40	0.00	11.20	184.8
C12	5.00	23.80	6.60	19.00	13.00	20.60	3.40	2.20	6.40	8.80	11.20	0.00	120

where: C1–C12 as in the causes list.

**Table A5:** Ordered matrix containing the total impact of mutual relations of potential causes in terms of quality and environment for the problem of porosity cluster on the mechanical seal made of alloy 410.

	C2	C1	C3	C4	C5	C6	C7	C8	C9	C10	C11	C12	Total Impact
C2	0.00	22.80	20.40	7.20	16.80	4.40	26.40	24.40	28.60	31.00	33.40	23.80	239.2
C1	22.80	0.00	2.40	18.00	8.00	18.40	8.40	7.20	11.40	13.80	16.20	5.00	131.6
C3	20.40	2.40	0.00	15.60	6.40	16.00	10.00	8.80	13.00	15.40	17.80	6.60	132.4
C4	7.20	18.00	15.60	0.00	12.00	5.60	21.60	18.80	21.40	23.80	26.20	19.00	189.2
C5	34.00	0.00	4.00	34.00	12.00	24.00	18.00	20.00	29.00	33.00	37.00	13.00	165.6
C6	4.40	18.40	16.00	5.60	12.40	0.00	24.00	22.80	27.00	29.40	31.80	20.60	212.4
C7	26.40	8.40	10.00	21.60	16.40	24.00	0.00	2.80	3.00	5.40	7.80	3.40	129.2
C8	24.40	7.20	8.80	18.80	15.20	22.80	2.80	0.00	4.20	6.60	9.00	2.20	122
C9	28.60	11.40	13.00	21.40	19.40	27.00	3.00	4.20	0.00	2.40	4.80	6.40	141.6
C10	31.00	13.80	15.40	23.80	21.80	29.40	5.40	6.60	2.40	0.00	2.40	8.80	160.8
C11	33.40	16.20	17.80	26.20	24.20	31.80	7.80	9.00	4.80	2.40	0.00	11.20	184.8
C12	23.80	5.00	6.60	19.00	13.00	20.60	3.40	2.20	6.40	8.80	11.20	0.00	120

where: C1–C12 as in the causes list.
